# Network-Assisted Prediction of Potential Drugs for Addiction

**DOI:** 10.1155/2014/258784

**Published:** 2014-02-09

**Authors:** Jingchun Sun, Liang-Chin Huang, Hua Xu, Zhongming Zhao

**Affiliations:** ^1^School of Biomedical Informatics, The University of Texas Health Science Center at Houston, Houston, TX 77030, USA; ^2^Department of Biomedical Informatics, Vanderbilt University School of Medicine, 2525 West End Avenue, Suite 600, Nashville, TN 37203, USA; ^3^Department of Psychiatry, Vanderbilt University School of Medicine, Nashville, TN 37212, USA; ^4^Department of Cancer Biology, Vanderbilt University School of Medicine, Nashville, TN 37232, USA

## Abstract

Drug addiction is a chronic and complex brain disease, adding much burden on the community. Though numerous efforts have been made to identify the effective treatment, it is necessary to find more novel therapeutics for this complex disease. As network pharmacology has become a promising approach for drug repurposing, we proposed to apply the approach to drug addiction, which might provide new clues for the development of effective addiction treatment drugs. We first extracted 44 addictive drugs from the NIDA and their targets from DrugBank. Then, we constructed two networks: an addictive drug-target network and an expanded addictive drug-target network by adding other drugs that have at least one common target with these addictive drugs. By performing network analyses, we found that those addictive drugs with similar actions tended to cluster together. Additionally, we predicted 94 nonaddictive drugs with potential pharmacological functions to the addictive drugs. By examining the PubMed data, 51 drugs significantly cooccurred with addictive keywords than expected. Thus, the network analyses provide a list of candidate drugs for further investigation of their potential in addiction treatment or risk.

## 1. Introduction

Drug addiction is a chronic and relapsing brain disease that causes compulsive drug seeking and abuse. The disease affects the brain functions and behavior of many people of all ages. The subjects suffer harmful consequences of drug addiction, which generates an enormous medical, financial, social, and emotional burden on individuals, their families, and our society. During the past several decades, investigators have made numerous efforts to understand the neuronal effects of addictive drugs and the molecular mechanisms of addiction. Such knowledge has facilitated the uncovering of novel targets and drugs for both treating and preventing addictive disorders.

The large body of studies has revealed that genetic and environmental factors contribute to the development of addiction [1]. The genetic studies of twins and families have suggested that genetic factors might account for 30–60% of the overall risk for the development of drug addiction [2, 3]. The recent advent of high-throughput experimental technologies, such as gene expression profiling, genome-wide association studies (GWAS), and next-generation sequencing (NGS), has revolutionized biomedical research and generated a massive amount of data for addiction research [4, 5]. This provides valuable information for further development of addiction treatment. Even so, an effective treatment of drug addiction patients is still unavailable.

Currently, medication and behavioral therapy, especially when combined, are the major therapeutic treatment approaches for addiction [6]. Thus, the discovery of effective drugs with fewer side effects is crucial to provide effective treatment and prevent relapse. During the past decade, advancements in target-based approaches have provided us with a promising direction for further treatment development [7]. Therefore, systematic investigations of addictive drugs and their targets might provide deeper insights into the relationship between individual addictive drugs and nonaddictive drugs. However, the absence of comprehensive drug-target data is a major limitation in performing a systematic investigation. Recently, DrugBank has provided a comprehensive collection of drugs and their targets [8], which largely eases this problem. Other drug-target databases have become available to assist further computational analyses [9, 10]. Furthermore, the concept of network medicine has been proposed and various approaches have been developed to assist with drug-drug and drug-target discovery [11–14]. We recently applied network pharmacology approach to exploring the features of antipsychotic and illicit drugs as well as their targets and found some interesting drug-target interaction features [13–15]. Here, we expanded our work to perform a systematic investigation of the relationships between multiple addictive drugs and their targets, as well as other drugs that have targets in common with these addictive drugs. The inclusion of addiction-related drugs might help us predict other addiction-related drugs from available drugs through drug repurposing approaches. We hypothesize that some of the addiction-related drugs that have not been assigned as addictive drugs might have the potential to treat addiction, while others might cause addictive effects.

We mainly focused on the addictive drugs annotated by the National Institute on Drug Abuse (NIDA). We extracted their targets from the DrugBank database. We first constructed a basic addictive drug-target network from which we attempted to find the unique connectivity as a proof of the concept of the network pharmacology approach for addictive drugs. Next, we built an expanded addictive drug-target network by recruiting non-addictive drugs. These non-addictive drugs have at least one target in common with at least one addictive drug. We analyzed these two networks by examining their network topological characteristics, which allowed us to explore whether some of the non-addictive drugs in the network might have the potential to either be addictive themselves or have the potential to treat addiction. Finally, to explore some lines of evidence from previous studies, we examined cooccurrence of drugs and addiction-related keywords to evaluate the association of non-addictive drugs with addiction. This preliminary study demonstrated that the network-assisted approach is promising in the prediction of drug repurposing.

## 2. Materials and Methods

### 2.1. Addictive Drugs and Drug Targets

In this study, we define addictive drugs as those abused drugs and prescribed drugs that can cause addiction disease once they are abused by humans. We manually obtained a list of the abused drugs from the Commonly Abused Drugs Chart and the prescribed drugs from the Prescription Drugs Abuse Chart created by the National Institute on Drug Abuse (NIDA) (http://drugabuse.gov/). The two charts contain addictive drugs along with their common and street names. These addictive drugs could be grouped into six categories according to similarities between how they work and what effects they produce in the human body, especially in the brain. These six categories are depressants, dissociative anesthetics, hallucinogens, opioids and morphine derivatives, stimulants, and other compounds.

We extracted the drug target data from DrugBank, a publically available database [8]. DrugBank includes 6712 drugs and 150 corresponding data fields for each drug. To match the addictive drugs collected from NIDA to DrugBank, we first manually searched the DrugBank website (http://www.drugbank.ca/) by using the drugs' common names and then collected their DrugBank accession numbers. The “Accession Number” is the unique DrugBank ID consisting of a two-letter prefix (DB) and a five-digit suffix. Next, we obtained their targets and the non-addictive drugs that share at least one target with at least one addictive drug from the DrugBank XML file (version 3.0) downloaded in July, 2013. We extracted the corresponding data from the following fields: “Name,” “Groups,” and “Targets.” The “Name” field includes the standard name of a drug as provided by the drug manufacturer. The “Groups” field represents the legal status of a drug such as “Approved,” “Experimental,” “Nutraceutical,” “Illicit,” and “Withdrawn” (detailed information can be found on the DrugBank website). The “Targets” field contains drug targets to which one drug can bind, including proteins, macromolecules, nucleic acids, or small molecules. In this study, we primarily extracted human proteins with UniProtKB identifiers and then mapped them to Entrez gene symbols and gene IDs using the UniProt ID mapping service (http://www.uniprot.org/mapping/).

### 2.2. Drug ATC Classification

To systematically examine drug classifications of addictive and non-addictive drugs, we further employed the Anatomical Therapeutic Chemical (ATC) classification (http://www.whocc.no/atc_ddd_index/). The classification system categorizes active drugs into five different levels based on the organ or system on which they act as well as their therapeutic and chemical characteristics. For each drug, the ATC classification information was extracted from the DrugBank XML file (version 3.0) or the Kyoto Encyclopedia of Genes and Genomes (KEGG) DRUG “htext” file, which was downloaded from KEGG Anatomical Therapeutic Chemical (ATC) classification website (http://www.genome.jp/kegg-bin/get_htext?br08303.keg) in July, 2013.

### 2.3. Functional Analysis of Targets

To characterize the functionality of those addictive drugs' targets, we performed an enrichment analysis of KEGG canonical pathways using the online tool Web-Based Gene Set Analysis Toolkit (WebGestalt) [16]. After the genes of interest were input into the WebGestalt system, it mapped the genes to the KEGG annotation and performed hypergeometric tests. To reduce the type I error, we conducted the Benjamini-Hochberg correction for multiple testings [17]. Using this approach, we calculated the adjusted *P* values to assess the overrepresentation of these input genes in each biological pathway. Here, we selected the pathways with adjusted *P*-values of less than 0.01 as the significantly enriched pathways. To further ensure a biologically meaningful analysis, we considered only those KEGG pathways that contained at least five target genes [18].

### 2.4. Network Construction, Visualization, and Analyses

We constructed two addiction-related networks. The first one is the addictive drug-target network, in which the nodes represent addictive drugs or their targets and edges represent the associations between these drugs and targets. The second network is an expanded addictive drug-target network, in which nodes include addictive drugs, their targets, and non-addictive drugs that have at least one target in common with addictive drugs. We employed the software Cytoscape (version 3.01) [19] to visualize and analyze the networks.

Considering that the nodes that act as hubs or bridging nodes in a network might play critical roles in drug actions [11, 20], we performed degree and betweenness analyses to determine the hubs and bridge nodes. In the network, a node with a higher degree (number of edges linked to the node) is defined as a hub. Hubs play important roles in biological networks because they tend to be encoded by essential genes [21]. In this study, we determine hubs by plotting the degree distribution, adopting the methods described by Yu et al. [22]. We defined the degree value as the cutoff where the distribution begins to straighten out. For bridging nodes, we calculated the betweenness centrality using algorithms implemented in the Cytoscape plugin NetworkAnalyzer [23] and then drew the betweenness distribution to define the point that the distribution began to reach its asymptote.

### 2.5. Literature Search

To evaluate the prediction of non-addictive drugs' associations with addiction, we adopted the NCBI PubMed automatic term mapping strategy to examine whether a drug and an addiction-related keyword cooccur in the same PubMed document [24]. The addiction-related keywords included “addiction,” “addictive,” “abuse,” and “abused.” The total number of abstract records in the 2012 PubMed was 21,508,439 (http://www.nlm.nih.gov/bsd/authors1.html). For each drug, we obtained three numbers corresponding to three subsets of PubMed abstracts: the number of abstracts with the given drug name, the number of abstracts with at least one addiction-related keyword, and the unique number of abstracts with a co-occurrence of the drug name and at least one of the addiction-related keywords. Then, we performed the Fisher's exact test based on these numbers for each drug. To identify and determine the predicted non-addictive drugs that were more significantly associated with addiction study than expected, we required that the drugs have a *P* value of less than 0.05 after Bonferroni multiple testing correction [17].

## 3. Results

### 3.1. Addictive Drugs and Their Targets

This study included 44 compounds listed as addictive drugs by NIDA. We extracted their target information from the DrugBank database. Among them, 39 belonged to the approved drugs category in at least one country, 22 were illicit drugs that were scheduled in at least one country, three were withdrawn drugs, and three were experimental drugs. According to similarities regarding how they function and what effects they produce in the human body and brain, as annotated by the NIDA, these drugs could be grouped into six categories: depressants (12), dissociative anesthetics (2), hallucinogens (1), opioids and morphine derivatives (10), stimulants (6), and other compounds (13).  Table 1 summarizes the detailed information for each drug.

According to ATC system classification, 32 drugs belonged to “nervous system,” four to “respiratory system,” three to “alimentary tract and metabolism,” and two to “sensory organs.” This observation confirmed that almost all of the addictive drugs perform their actions by affecting brain function.

Among the 44 addictive drugs, 41 had at least one target gene. After deleting redundancy and mapping gene names to NCBI gene annotations (http://www.ncbi.nlm.nih.gov/gene), we obtained 91 target genes (additional file, Table S1 in supplementary material available online at http://10.1155/2014/258784). To examine the pathways in which those target genes involve, we conducted a KEGG pathway enrichment analysis using the online tool WebGestalt. Nine pathways were significantly enriched with the 91 addictive drug target genes (adjusted *P*-value < 0.01) (Table 2). Among them, the most significant one is “neuroactive ligand-receptor interaction,” which includes more than half of the addictive drug target genes (61.54%). This pathway finding is consistent with the molecular mechanisms underlying the addiction [25].

### 3.2. Addictive Drug-Target Network

**Figure 1:**
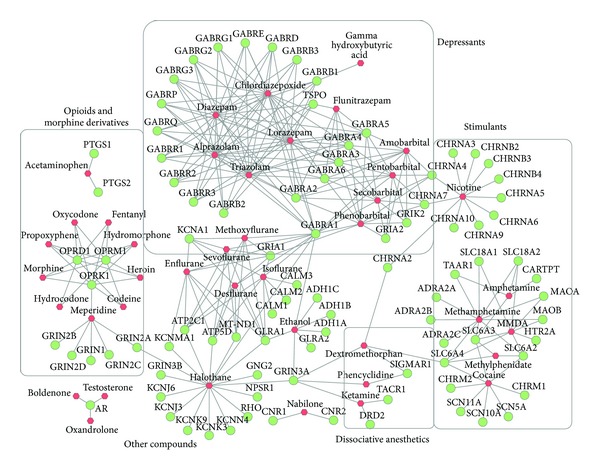
The addictive drug-target network. The red nodes denote addictive drugs and the green nodes denote their targets. The edges indicate the relationship between each drug and its targets. Subnetworks are highlighted to differentiate five drug categories: depressants, stimulants, opioids and morphine derivatives, dissociative anesthetics, and other compounds.

According to the relationship between addictive drugs and their targets, we first generated an addictive drug-target interaction network, which provided general insights into the organization and association between addictive drugs and their targets. Through finding interesting features from this network, we aimed to prove the value of the network application concept when investigating drug repurposing. In this network, an addictive drug connects to a target (i.e., an edge) if the target is a known target of the drug. The addictive drug-target network contained 132 nodes (41 addictive drugs and 91 target genes) and 297 edges. After superimposing the drug categories onto the network, five clusters were observed, which corresponded to five major drug categories: depressants, stimulants, dissociative anesthetics, opioids and morphine derivatives, and other compounds (Figure 1). Interestingly, there are several bridging nodes that link the major subnetworks together. These bridging nodes are GABRA1, SLC6A4, GRIN3A, CHRNA2, CHRNA4, and CHRNA7.

### 3.3. Expanded Addictive Drug-Target Interaction Network

Drugs sharing the same targets might participate in the same pathways and have similar actions. Thus, an investigation of the drugs that share the same targets with addictive drugs might provide information for further addiction treatment. Here, we added these non-addictive drugs to the addictive drug-target network to construct an expanded addictive drug-target interaction network. The network contained 705 nodes and 1797 edges. These 705 nodes included 41 addictive drugs, 573 non-addictive drugs, and 91 targets. The edges contained 297 interactions between addictive drugs and their targets and 1500 interactions between non-addictive drugs and addictive drug targets.

Among these 573 non-addictive drugs, 407 had at least one ATC classification distributed among all 14 categories. Among them, the percentage of addictive and non-addictive drugs was significantly different in the category of “nervous system” (N) (Fisher's exact test, *P*-value: 2.56 × 10^−5^). Though almost half of the non-addictive drugs (203/407, 49.88%) belong to “nervous system,” this proportion is significantly lower than that of addictive drugs involved in the expanded network (32/38, 84.21%; *P*-value: 3.00 × 10^−5^). The difference in the category “nervous system” was expected since almost all addictive drugs function through the brain.

**Figure 2:**
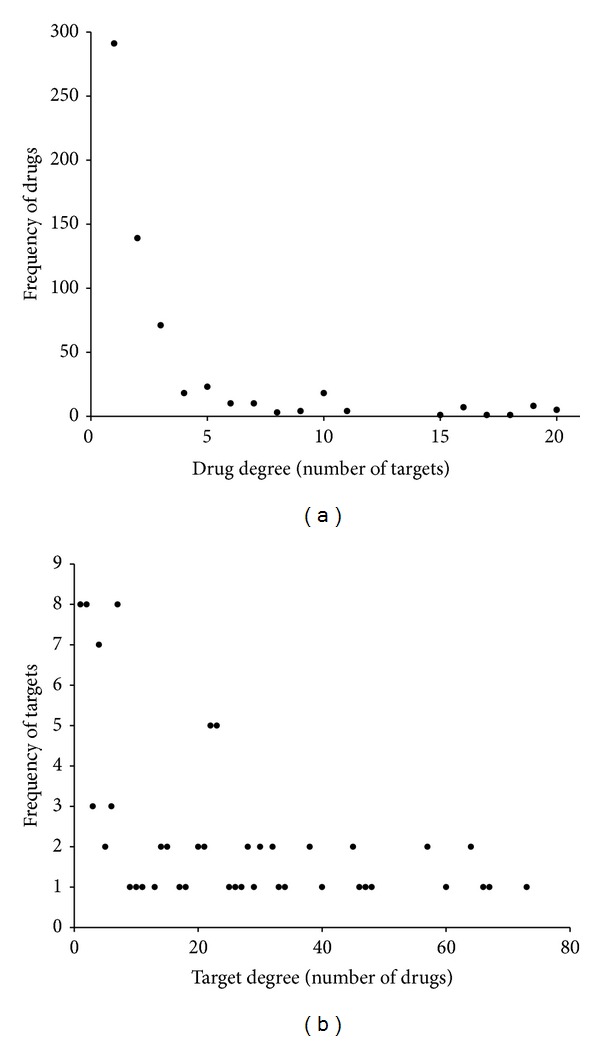
Degree distribution of drugs (a) and targets (b) in the expanded addictive drug-target network.

**Figure 3:**
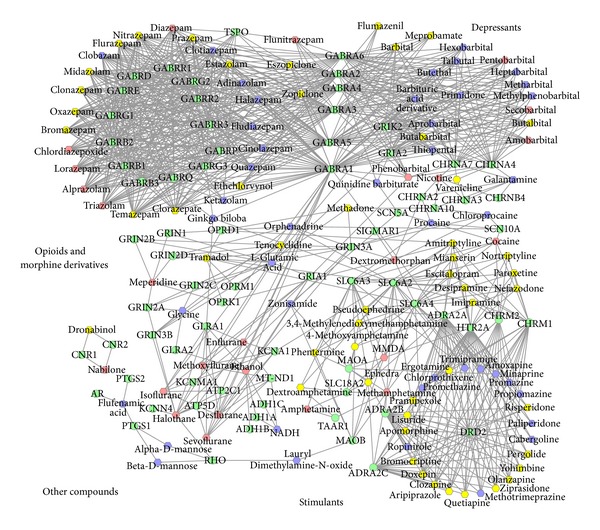
The expanded addictive drug-target network after filtering by hubs and bridge nodes. The red nodes denote addictive drugs, the green nodes denote targets, the yellow nodes denote nonaddictive drugs that have a significantly higher cooccurrence than expected with addiction-related keywords in the literature from PubMed, and the blue nodes denote non-addictive drugs that have a co-occurrence with addiction keywords but are not significantly higher than expected in the literature data.

In the network, the average drug degree (number of targets) was 2.9 with a range between 1 and 20, while the average target degree (number of drugs) was 19.5 with a range between 1 and 73. In the network, the target degree was oversaturated compared to the drug degree, which was mainly caused by the approach used to generate this network. The distribution of drug degrees followed a power law, but the distribution for target degrees did not have this feature (Figure 2). Thus, to identify the drugs related to addiction, we calculated the drug degree distribution to determine the drug hubs in the network. As shown in Figure 2, the nodes with degrees greater than three were defined as hubs. Similarly, we defined each node with a betweenness centrality greater than 0.04 as a bridging node (data not shown). After retaining the hub nodes and bridging nodes, a subnetwork was extracted from the expanded addictive drug-target network. The subnetwork contained 193 nodes (25 addictive drugs, 94 non-addictive drugs, and 74 targets) and 1002 edges (Figure 3). As a result, we identified 94 drugs that either have a high potential for having addictive effects or could be used as a potential treatment for addiction. The degree and betweenness values of these 94 drugs were provided in additional file, Table S2.

### 3.4. Evaluation of Predicted Non-Addictive Drugs for Addiction

To evaluate the association between these 94 non-addictive drugs and addiction, we examined the co-occurrence of each drug and addiction-related keywords including “addiction,” “addictive,” “abuse,” and “abused” in PubMed abstracts. Among the 94 drugs, 51 drugs (54.26%) (yellow nodes in Figure 3) had statistically significant *P*-values after Bonferroni correction of multiple testing (Fisher's exact test, *P*-value: 0 ~ 0.0004) (additional file, Table S2). For example, the drug temazepam, which is a hub with 20 targets shared with addictive drugs, is a highly addictive benzodiazepine medication [26–30]. The drug dronabinol, which is the strongest drug bridge node in the network, has the potential for addiction [31]. It is also a promising medication for the treatment of cannabis dependence [32]. The drug methadone is the fourteenth strongest bridge node and the top one based on the ratio of the observed versus expected number of documents in PubMed. We added more discussion below.

**Figure 4:**
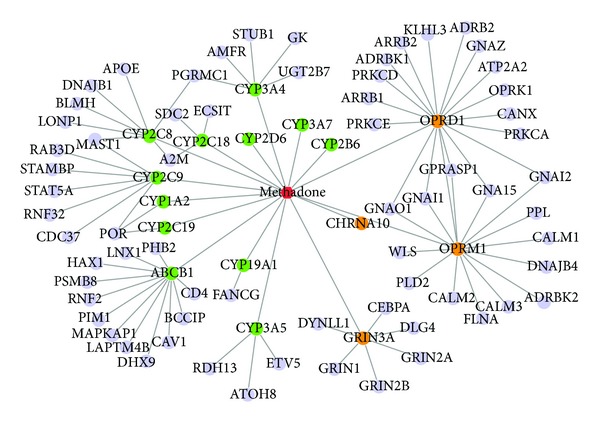
The methadone drug-target network. The red nodes denote the methadone (drug), the orange nodes denote its targets, the green nodes denote its enzymes, and the blue nodes denote the directly interacting proteins of targets and enzymes from protein-protein interaction network.

Methadone is the most widely available pharmacotherapy for opioid addiction and it has been shown to be an effective and safe treatment for many years [33, 34]. To illustrate the molecular mechanism of this drug, we generated a methadone-specific network (Figure 4). This network included 74 nodes and 94 edges. The nodes included the drug methadone, its four targets and 12 enzymes from DrugBank, and 67 proteins directly interacting with the four targets and 12 enzymes (Figure 4). The edges included 4 interactions between the drug and four targets, 12 relationship between the drug and 12 enzymes, and 78 protein-protein interactions between targets/enzymes and other proteins, which were extracted from the protein interaction network analysis (PINA) database [35]. According to the KEGG pathway annotations, all four targets (OPRM1, GRIN3A, CHRNA10, and OPRD1) are neuroactive ligand receptors. Among the 12 enzymes, ten are directly involved in drug metabolism. There are 20 KEGG pathways that were significantly enriched in the 67 proteins. Among them, seven pathways are directly involved in the neurodevelopment, including “Long-term potentiation” (7 proteins: CALM2, CALM3, GRIN2A, PRKCA, CALM1, GRIN2B, and GRIN1; *P*-value: 1.75 × 10^−11^), “Long-term depression” (5 proteins: PRKCA, GNAI2, GNAZ, GNAO1, and GNAI1; *P*-value: 8.70 × 10^−8^), and “Neuroactive ligand-receptor interaction” (5 proteins: ADRB2, OPRK1, GRIN2A, GRIN2B, and GRIN1; *P*-value: 6.75 × 10^−5^). These observations confirmed that methadone directly acts with neurotransmitters and further regulates the other molecular components in neurodevelopment.

Put together, the drug pool through our network analyses might provide a list of candidate drugs for further investigation of their potential for addiction treatment or addiction risk.

## 4. Discussion

In this study, we investigated the relationships between addictive drugs, their targets, and non-addictive drugs that have targets in common with addictive drugs in the context of drug-target networks. Most of the addictive drugs with similar functions could cluster together in their drug-target network (Figure 1), indicating that network-assisted approaches could effectively capture drug classification characteristics. After studying the network topological characteristics, we predicted some drugs that might have the potential leading to addictive effects or to addiction treatment. These results illustrate that the network pharmacology approach is promising for drug repositioning [36, 37]. Therefore, the strategy employed for building the basic and the expanded networks in this study is effective and straightforward, offering a promising computational method to predict potential drugs for a given disease. Furthermore, this study proves the concept that such a network approach can be implemented in predicting drug-target relationships and uncovering novel drugs/targets for both basic and clinical research.

We mainly extracted the drugs and their targets from DrugBank. Though the study provides some promising results, future improvement is needed. One limitation of this study is that the current data is neither complete nor bias-free. In future, we will include more drug-target information from multiple data sources such as the binding database (binding DB) [38], therapeutic targets database (TTD) [39], and other drug-target centered databases. We also expect that data quality and annotations of drug-target interactions will be substantially improved in the near future due to numerous ongoing efforts in this research area.

In our previous study, we explored the relationship between illicit drugs and their targets [15]. Illicit drugs are those drugs that are annotated as illicit in at least one country according to DrugBank annotation. Some illicit drugs could lead to addiction once they are abused by humans. However, only the drugs that could lead to addiction are referred to as abused drugs by NIDA. In this study, we mainly focused on the 44 drugs that lead to addiction, of which only 20 belong to the illicit drugs category.

In this study, we predicted 94 non-addictive drugs that might have associations with addiction. To explore if some of these drugs have been studied with addiction, we used a keyword-based literature search followed by a co-occurrence analysis. The literature search approach we utilized largely relied on the co-occurrence of addictive drugs and addiction-related keywords in the PubMed database. The high throughput literature search revealed that more than half (54.26%) of non-addictive drugs have been previously investigated or reported as linked to addiction. However, the current literature survey method did not allow us to examine the logical relationship between these drugs and addiction. Thus, we could not filter those negative studies based on negative logical relationship information in abstracts. In the future, we may improve our strategy for searching the co-occurrence of drugs and keywords by creating a more efficient algorithm using natural language processing techniques.

## Supplementary Material

The Supplementary Materials for this paper include two Tables. Table S1 provides the list of 91 target genes of 44 addictive drugs. Table S2 includes the non-addictive drugs involved in the expanded addictive drug-target network, their network properties (degree and betweenness), and literature search evaluation.

## Figures and Tables

**Table 1:** Summary of addictive drugs, their targets, and classification.

DrugBank ID	Drug name	Number of targets	DrugBank group	NIDA category^a^
DB00316	Acetaminophen	2	Approved	Opioids and morphine derivatives
DB00404	Alprazolam	20	Approved, illicit	Depressants
DB01351	Amobarbital	10	Approved, illicit	Depressants
DB00182	Amphetamine	4	Approved, illicit	Stimulants
DB01541	Boldenone	1	Experimental, illicit	Other compounds
DB00475	Chlordiazepoxide	19	Approved, illicit	Depressants
DB00907	Cocaine	8	Approved, illicit	Stimulants
DB00318	Codeine	3	Approved, illicit	Opioids and morphine derivatives
DB01189	Desflurane	7	Approved	Other compounds
DB00514	Dextromethorphan	4	Approved	Other compounds
DB00829	Diazepam	18	Approved, illicit	Depressants
DB00228	Enflurane	8	Approved	Other compounds
DB00898	Ethanol	7	Approved	Other compounds
DB00813	Fentanyl	3	Approved, illicit	Opioids and morphine derivatives
DB01544	Flunitrazepam	6	Approved, illicit	Depressants
DB01440	Gamma hydroxybutyric acid	1	Approved, illicit	Depressants
DB01159	Halothane	17	Approved	Other compounds
DB01452	Heroin	3	Approved, illicit	Opioids and morphine derivatives
DB00956	Hydrocodone	2	Approved, illicit	Opioids and morphine derivatives
DB00327	Hydromorphone	3	Approved, illicit	Opioids and morphine derivatives
DB00753	Isoflurane	7	Approved	Other compounds
DB01221	Ketamine	3	Approved	Dissociative anesthetics
DB00186	Lorazepam	20	Approved	Depressants
DB04829	Lysergic acid diethylamide	0	Illicit, withdrawn	Hallucinogens
DB00454	Meperidine	6	Approved	Opioids and morphine derivatives
DB01577	Methamphetamine	11	Approved, illicit	Stimulants
DB04833	Methaqualone	0	Illicit, withdrawn	Depressants
DB01028	Methoxyflurane	7	Approved	Other compounds
DB00422	Methylphenidate	3	Approved, investigational	Stimulants
DB01442	MMDA	8	Experimental, illicit	Stimulants
DB00295	Morphine	3	Approved	Opioids and morphine derivatives
DB00486	Nabilone	2	Approved	Other compounds
DB00984	Nandrolone phenpropionate	0	Approved, illicit	Other compounds
DB00184	Nicotine	11	Approved	Stimulants
DB00621	Oxandrolone	1	Approved	Other compounds
DB00497	Oxycodone	3	Approved, illicit	Opioids and morphine derivatives
DB00312	Pentobarbital	10	Approved	Depressants
DB03575	Phencyclidine	2	Experimental, illicit	Dissociative anesthetics
DB01174	Phenobarbital	10	Approved	Depressants
DB00647	Propoxyphene	3	Approved, illicit	Opioids and morphine derivatives
DB00418	Secobarbital	10	Approved	Depressants
DB01236	Sevoflurane	7	Approved	Other compounds
DB00624	Testosterone	1	Approved	Other compounds
DB00897	Triazolam	20	Approved, illicit, withdrawn	Depressants

^a^Drug category is defined based on the similarities regarding how drugs function and what effects they produce in the human body, including the brain, as annotated by NIDA.

**Table 2:** KEGG pathways significantly enriched with the target genes of addictive drugs.

Pathway name	Number of target genes (%)	Nominal *P* value^a^	Adjusted *P* value^b^
Neuroactive ligand-receptor interaction	56 (61.54)	8.65 × 10^−102^	7.78 × 10^−101^
Long-term potentiation	10 (10.99)	3.35 × 10^−16^	1.51 × 10^−15^
Calcium signaling pathway	12 (13.19)	3.25 × 10^−15^	9.75 × 10^−15^
Amyotrophic lateral sclerosis (ALS)	7 (7.69)	1.94 × 10^−11^	4.36 × 10^−11^
Alzheimer's disease	9 (9.89)	8.50 × 10^−11^	1.53 × 10^−10^
Tyrosine metabolism	5 (5.49)	2.50 × 10^−8^	3.75 × 10^−8^
Drug metabolism-cytochrome P450	5 (5.49)	4.75 × 10^−7^	6.11 × 10^−7^
Salivary secretion	5 (5.49)	1.28 × 10^−6^	1.44 × 10^−6^
Metabolic pathways	8 (8.79)	2.50 × 10^−3^	2.50 × 10^−3^

^a^Nominal *P* values were calculated using the hypergeometric test.

^b^Adjusted *P* values were estimated by Benjamini-Hochberg (1995) multiple testing corrections [17].
