# Supplementary material for: Network-Assisted Prediction of Potential Drugs for Addiction
Source: Biomed Res Int. 2014 Feb 9;2014:258784. doi: 10.1155/2014/258784 (PMC3932722; doi:10.1155/2014/258784)
Supplement: Supplementary file 1 — The Supplementary Materials for this paper include two Tables. Table S1 provides the list of 91 target genes of 44 addictive drugs. Table S2 includes the non-addictive drugs involved in the expanded addictive drug-target network, their network properties (degree and betweenness), and literature search evaluation. [file 258784.f1.pdf]

Additional file

Table S1. 91 target genes by addictive drugs

Symbol	Gene ID	Name
ADH1A	124	alcohol dehydrogenase 1A (class I), alpha polypeptide
ADH1B	125	alcohol dehydrogenase 1B (class I), beta polypeptide
ADH1C	126	alcohol dehydrogenase 1C (class I), gamma polypeptide
ADRA2A	150	adrenoceptor alpha 2A
ADRA2B	151	adrenoceptor alpha 2B
ADRA2C	152	adrenoceptor alpha 2C
AR	367	androgen receptor
ATP2C1	27032	ATPase, Ca++ transporting, type 2C, member 1
ATP5D	513	ATP synthase, H+ transporting, mitochondrial F1 complex, delta subunit
CALM1	801	calmodulin 1 (phosphorylase kinase, delta)
CALM2	805	calmodulin 2 (phosphorylase kinase, delta)
CALM3	808	calmodulin 3 (phosphorylase kinase, delta)
CARTPT	9607	CART prepropeptide
CHRM1	1128	cholinergic receptor, muscarinic 1
CHRM2	1129	cholinergic receptor, muscarinic 2
CHRNA10	57053	cholinergic receptor, nicotinic, alpha 10 (neuronal)
CHRNA2	1135	cholinergic receptor, nicotinic, alpha 2 (neuronal)
CHRNA3	1136	cholinergic receptor, nicotinic, alpha 3 (neuronal)
CHRNA4	1137	cholinergic receptor, nicotinic, alpha 4 (neuronal)
CHRNA5	1138	cholinergic receptor, nicotinic, alpha 5 (neuronal)
CHRNA6	8973	cholinergic receptor, nicotinic, alpha 6 (neuronal)
CHRNA7	1139	cholinergic receptor, nicotinic, alpha 7 (neuronal)
CHRNA9	55584	cholinergic receptor, nicotinic, alpha 9 (neuronal)
CHRNB2	1141	cholinergic receptor, nicotinic, beta 2 (neuronal)
CHRNB3	1142	cholinergic receptor, nicotinic, beta 3 (neuronal)
CHRNB4	1143	cholinergic receptor, nicotinic, beta 4 (neuronal)
CNR1	1268	cannabinoid receptor 1 (brain)
CNR2	1269	cannabinoid receptor 2 (macrophage)
DRD2	1813	dopamine receptor D2
GABRA1	2554	gamma-aminobutyric acid (GABA) A receptor, alpha 1
GABRA2	2555	gamma-aminobutyric acid (GABA) A receptor, alpha 2
GABRA3	2556	gamma-aminobutyric acid (GABA) A receptor, alpha 3
GABRA4	2557	gamma-aminobutyric acid (GABA) A receptor, alpha 4
GABRA5	2558	gamma-aminobutyric acid (GABA) A receptor, alpha 5

GABRA6	2559	gamma-aminobutyric acid (GABA) A receptor, alpha 6
GABRB1	2560	gamma-aminobutyric acid (GABA) A receptor, beta 1
GABRB2	2561	gamma-aminobutyric acid (GABA) A receptor, beta 2
GABRB3	2562	gamma-aminobutyric acid (GABA) A receptor, beta 3
GABRD	2563	gamma-aminobutyric acid (GABA) A receptor, delta
GABRE	2564	gamma-aminobutyric acid (GABA) A receptor, epsilon
GABRG1	2565	gamma-aminobutyric acid (GABA) A receptor, gamma 1
GABRG2	2566	gamma-aminobutyric acid (GABA) A receptor, gamma 2
GABRG3	2567	gamma-aminobutyric acid (GABA) A receptor, gamma 3
GABRP	2568	gamma-aminobutyric acid (GABA) A receptor, pi
GABRQ	55879	gamma-aminobutyric acid (GABA) A receptor, theta
GABRR1	2569	gamma-aminobutyric acid (GABA) A receptor, rho 1
GABRR2	2570	gamma-aminobutyric acid (GABA) A receptor, rho 2
GABRR3	200959	gamma-aminobutyric acid (GABA) A receptor, rho 3
GLRA1	2741	glycine receptor, alpha 1
GLRA2	2742	glycine receptor, alpha 2
GNG2	54331	guanine nucleotide binding protein (G protein), gamma 2
GRIA1	2890	glutamate receptor, ionotropic, AMPA 1
GRIA2	2891	glutamate receptor, ionotropic, AMPA 2
GRIK2	2898	glutamate receptor, ionotropic, kainate 2
GRIN1	2902	glutamate receptor, ionotropic, N-methyl D-aspartate 1
GRIN2A	2903	glutamate receptor, ionotropic, N-methyl D-aspartate 2A
GRIN2B	2904	glutamate receptor, ionotropic, N-methyl D-aspartate 2B
GRIN2C	2905	glutamate receptor, ionotropic, N-methyl D-aspartate 2C
GRIN2D	2906	glutamate receptor, ionotropic, N-methyl D-aspartate 2D
GRIN3A	116443	glutamate receptor, ionotropic, N-methyl-D-aspartate 3A
GRIN3B	116444	glutamate receptor, ionotropic, N-methyl-D-aspartate 3B
HTR2A	3356	5-hydroxytryptamine (serotonin) receptor 2A, G protein-coupled
KCNA1	3736	potassium voltage-gated channel, shaker-related subfamily, member 1 (episodic ataxia with myokymia)
KCNJ3	3760	potassium inwardly-rectifying channel, subfamily J, member 3
KCNJ6	3763	potassium inwardly-rectifying channel, subfamily J, member 6
KCNK3	3777	potassium channel, subfamily K, member 3
KCNK9	51305	potassium channel, subfamily K, member 9
KCNMA1	3778	potassium large conductance calcium-activated channel, subfamily M, alpha member 1
KCNN4	3783	potassium intermediate/small conductance calcium-activated channel, subfamily N, member 4
MAOA	4128	monoamine oxidase A
MAOB	4129	monoamine oxidase B
MT-ND1	4535	NADH dehydrogenase, subunit 1 (complex I)
NPSR1	387129	neuropeptide S receptor 1
OPRD1	4985	opioid receptor, delta 1

OPRK1	4986	opioid receptor, kappa 1
OPRM1	4988	opioid receptor, mu 1
PTGS1	5742	prostaglandin-endoperoxide synthase 1 (prostaglandin G/H synthase and cyclooxygenase)
PTGS2	5743	prostaglandin-endoperoxide synthase 2 (prostaglandin G/H synthase and cyclooxygenase)
RHO	6010	rhodopsin
SCN10A	6336	sodium channel, voltage-gated, type X, alpha subunit
SCN11A	11280	sodium channel, voltage-gated, type XI, alpha subunit
SCN5A	6331	sodium channel, voltage-gated, type V, alpha subunit
SIGMAR1	10280	sigma non-opioid intracellular receptor 1
SLC18A1	6570	solute carrier family 18 (vesicular monoamine), member 1
SLC18A2	6571	solute carrier family 18 (vesicular monoamine), member 2
SLC6A2	6530	solute carrier family 6 (neurotransmitter transporter, noradrenalin), member 2
SLC6A3	6531	solute carrier family 6 (neurotransmitter transporter, dopamine), member 3
SLC6A4	6532	solute carrier family 6 (neurotransmitter transporter, serotonin), member 4
TAAR1	134864	trace amine associated receptor 1
TACR1	6869	tachykinin receptor 1
TSPO	706	translocator protein (18kDa)

Table S2. The network properties and literature search results of non-addictive drugs

**Drug name**	**Expanded addictive network**		**Literature search evaluation**		
	**Degree**	**Betweenness**	**Number of abstracts**[a]	**Number of abstracts**[b]	**Fisher's exact *P*-value**
3,4-Methylenedioxy methamphetamine	5	0.0041	590	1834	0
Aripiprazole	7	0.0045	91	2225	0
Bromazepam	19	0.0025	43	500	0
Desipramine	5	0.0032	188	7412	0
Dextroamphetamine	4	0.0018	417	6578	0
Dronabinol	2	0.1667	30	189	0
Ephedra	10	0.0280	36	683	0
Ergotamine	5	0.0025	101	2807	0
Ethchlorvynol	9	0.0012	23	246	0
Flurazepam	19	0.0025	37	1131	0
Meprobamate	6	0.0003	121	2466	0
Nitrazepam	19	0.0025	50	1201	0
Nortriptyline	5	0.0032	64	2720	0
Olanzapine	7	0.0045	185	6493	0
Oxazepam	19	0.0025	119	1723	0
Paroxetine	5	0.0032	134	5056	0

Phentermine	5	0.0103	58	844	0
Pramipexole	5	0.0007	33	1004	0
Pseudoephedrine	4	0.0014	82	1152	0
Risperidone	5	0.0007	170	7288	0
Temazepam	20	0.0030	100	964	0
Varenicline	4	0.0017	191	887	0
Zopiclone	5	0.0006	87	847	0
Butalbital	10	0.0037	17	126	8.70×10^{-14}
4-Methoxyamphetamine	6	0.0151	15	86	3.82×10^{-13}
Clorazepate	20	0.0030	21	441	2.45×10^{-12}
Tramadol	6	0.0463	210	3020	2.51×10^{-11}
Amitriptyline	9	0.0517	103	7922	4.23×10^{-11}
Methadone	4	0.0192	5328	12684	1.40×10^{-10}
Quetiapine	7	0.0045	129	3229	2.38×10^{-10}
Clozapine	7	0.0045	235	9594	2.68×10^{-10}
Clonazepam	19	0.0029	103	3658	2.98×10^{-10}
Flumazenil	4	0.0002	89	4005	3.58×10^{-10}
Apomorphine	5	0.0007	153	11082	3.98×10^{-10}
Doxepin	9	0.0101	30	1238	1.04×10^{-9}
Midazolam	19	0.0025	119	10570	5.19×10^{-9}
Tenocyclidine	6	0.0019	13	270	2.77×10^{-8}
Eszopiclone	5	0.0006	10	174	2.14×10^{-7}
Escitalopram	4	0.0020	25	1203	4.25×10^{-7}
Imipramine	5	0.0032	124	12204	5.16×10^{-7}
Nefazodone	5	0.0026	18	691	7.55×10^{-7}
Ziprasidone	7	0.0045	27	1516	2.74×10^{-6}
Bromocriptine	5	0.0007	88	8422	7.46×10^{-6}
Yohimbine	5	0.0007	88	8603	1.60×10^{-5}
Lisuride	5	0.0007	19	952	1.68×10^{-5}
Barbital	10	0.0037	28	1827	2.72×10^{-5}
Mianserin	4	0.0015	39	3038	4.48×10^{-5}
Estazolam	16	0.0020	7	156	6.91×10^{-5}
Pergolide	5	0.0007	18	1103	3.28×10^{-4}
Prazepam	16	0.0020	7	208	4.04×10^{-4}
Butabarbital	10	0.0037	4	55	4.21×10^{-4}
Methotrimeprazine	7	0.0045	13	722	8.72×10^{-4}
Ropinirole	5	0.0007	12	652	1.13×10^{-3}
Halazepam	16	0.0020	3	57	5.73×10^{-3}
Fludiazepam	16	0.0020	2	27	0.01
Paliperidone	5	0.0007	6	375	0.03
Orphenadrine	6	0.0221	8	588	0.04
Promethazine	4	0.0018	30	3516	0.07
Promazine	4	0.0018	13	1284	0.07

Propiomazine	4	0.0018	2	68	0.07
Amoxapine	6	0.1018	5	426	0.14
Adinazolam	16	0.0020	2	108	0.15
Trimipramine	6	0.0048	5	469	0.18
Ketazolam	6	0.0009	1	34	0.19
Clobazam	19	0.0025	6	655	0.24
Galantamine	11	0.0148	12	1553	0.28
Clotiazepam	16	0.0020	1	55	0.29
Chloroprocaine	4	0.0124	3	417	0.49
Cabergoline	5	0.0007	7	1124	0.56
Quazepam	15	0.0018	1	143	0.60
Chlorprothixene	4	0.0018	3	660	0.79
Zonisamide	4	0.0135	4	961	0.86
Primidone	10	0.0037	7	1648	0.90
Flufenamic Acid	3	0.1088	2	1267	1.00
Procaine	4	0.0186	53	12524	1.00
Hexobarbital	10	0.0037	6	2706	1.00
Thiopental	10	0.0037	27	8039	1.00
Alpha-D-Mannose	2	0.1403	0	279	1.00
Aprobarbital	10	0.0037	0	19	1.00
Barbituric acid derivative	10	0.0037	0	23	1.00
Beta-D-Mannose	2	0.1403	0	39	1.00
Butethal	10	0.0037	0	4	1.00
Cinolazepam	16	0.0020	0	5	1.00
Ginkgobiloba	5	0.0268	0	3	1.00
Glycine	5	0.0010	216	62562	1.00
Heptabarbital	10	0.0037	0	31	1.00
Lauryl Dimethylamine-N-Oxide	2	0.0692	0	7	1.00
L-Glutamic Acid	10	0.0199	0	2301	1.00
Metharbital	10	0.0037	0	19	1.00
Methylphenobarbital	10	0.0037	0	47	1.00
Minaprine	5	0.0088	0	112	1.00
NADH	4	0.0051	36	29140	1.00
Talbutal	10	0.0037	0	3	1.00
Quinidine barbiturate	4	0.0322	1	0	1.00

[a]The number of abstracts based on having both the drug name and at least one addiction-related keyword in an abstract.
[b]The number of abstracts is based on the drug name in an abstract.
